# Supplementary material for: MetaRibo-Seq measures translation in microbiomes
Source: Nat Commun. 2020 Jun 29;11:3268. doi: 10.1038/s41467-020-17081-z (PMC7324362; doi:10.1038/s41467-020-17081-z)
Supplement: Supplementary file 10 — Supplementary Data 7 [file 41467_2020_17081_MOESM10_ESM.zip › File2/Confidence_VeryHigh_Taxonomy/279290_out.krona.html]

Javascript must be enabled to view this page.

members
magnitude
magnitudeUnassigned
count
unassigned
taxon
rank

279290\_out

15

1

SRS015782\_contig\_number\_contig-100\_29175.29176

2
14
superkingdom

7
976
phylum

200643
7
class

7
171549
order

family
7
171552

species
7
2049047

SRS011405\_contig\_number\_15140SRS016335\_contig\_number\_25322SRS021948\_contig\_number\_34384SRS024009\_contig\_number\_contig-100\_1501.62766SRS098073\_contig\_number\_12123SRS104084\_contig\_number\_6665SRS148319\_contig\_number\_11500

1239
7
phylum

7
186801
class

186802
7
order

186806
6
family

genus
6
1730

165185
1

SRS065504\_contig\_number\_25199
species


SRS014613\_contig\_number\_10732SRS047014\_contig\_number\_10598SRS054905\_contig\_number\_14943SRS142890\_contig\_number\_42752
4
39485
species


SRS147271\_contig\_number\_26269
1
142586
species

family
1
186803

1
39491

SRS893252\_contig\_number\_6794
species
